# Effective Control of Postprandial Glucose Level through Inhibition of Intestinal Alpha Glucosidase by *Cymbopogon martinii* (Roxb.)

**DOI:** 10.1155/2012/372909

**Published:** 2011-07-07

**Authors:** Varsha Ghadyale, Shrihari Takalikar, Vivek Haldavnekar, Akalpita Arvindekar

**Affiliations:** ^1^Department of Biochemistry, Shivaji University, Kolhapur 416 004, India; ^2^Ayurveda Clinic, Kolhapur 416008, India

## Abstract

Inhibition of intestinal alpha glucosidase plays a major role in preventing rise in postprandial glucose level in diabetics. *Cymbopogon martinii* (CM) (family Poaceae) is used in traditional Indian medicine in treatment of diabetes mellitus. The alpha glucosidase inhibitory action of the plant is studied. The active component was separated using hot water extraction of the whole plant powder, differential solvent extraction, and silica gel column chromatography. The 30 : 70 toluene : ethyl acetate fraction showed optimum activity. The silica gel chromatography fraction demonstrated 98, 98, and 68% inhibition for starch, maltose, and sucrose, respectively, at 5 mg/kg body weight of rats. Intestinal absorption studies using noneverted intestinal sacs, as well as in vivo studies in streptozotocin-induced diabetic rats using oral glucose tolerance with maltose and sucrose load, revealed better inhibition of alpha glucosidase as compared to acarbose. Kinetic studies using Lineweaver Burk plot showed mixed to noncompetitive type of inhibition by CM. In vivo studies with maltose load of 2 mg and 3 mg/gm body weight showed a noncompetitive pattern of inhibition at 5 mg/kg body weight of CM as against 60 mg/kg body weight of acarbose. Thus CM is more effective alpha glucosidase inhibitor and at lower concentration than acarbose.

## 1. Introduction

Diabetes mellitus is a progressive metabolic disorder of glucose metabolism that eventually leads to micro- and macrovascular changes causing secondary complications that are difficult to manage [1]. The control of hyperglycemia is therefore of prime importance to halt the progression of the disease. While use of insulin, secretagogues, and sensitizers constitutes the predominant line of therapy, use of inhibitors of intestinal absorption of sugar is vital as they do not interfere with the sugar metabolism and help control hyperglycemia in a noninvasive manner. The alpha glucosidases are exoglycosidases found on the luminal surface of enterocytes containing maltase/glucoamylase and sucrase/isomaltase activity [2]. Alpha glucosidase inhibitor, acarbose, is shown to control postprandial hyperglycemic shoot up [3] and is safe and well tolerated [4]. Alpha glucosidase inhibitors are useful as they reduce the cardiovascular risk [1]. 

*Cymbopogon martinii *(CM) also known as Rosha Grass is mainly used in perfume industry. The plant is used traditionally in treatment of diabetes. The plant has also been documented in Ayurveda for treatment of urinary tract infection, as anti-inflammatory and as diuretic [5]. In the present study *Cymbopogon martinii *(CM) belonging to the poaceae (Gramineae) family has been evaluated for its antidiabetic action through inhibition of alpha glucosidase in streptozotocin-induced diabetic rats and by evaluation of intestinal transport of sucrose and maltose.

## 2. Materials and Methods

### 2.1. Chemicals and Reagents

Streptozotocin, *α*-amylase from *Aspergillus oryzae*, alpha glucosidase from *Saccharomyces cerevisiae*, and *p*-Nitrophenyl *α*-D-glucopyranoside (PNPG) were purchased from Sigma Chemicals, USA. All other chemicals used for study were of AR grade and purchased from Qualigens India Pvt. Ltd., Mumbai and locally.

### 2.2. Plant Material

*Cymbopogon martinii *(Roxb.) Wats was collected from Kolhapur District, Maharashtra State, India and identified by the Botany Department of Shivaji University, Kolhapur, India. The herbarium specimen has been deposited in the Botany Department of Shivaji University (Voucher specimen number VAG 19 for CM). The whole plants, except roots, were air-dried in shade and grounded into powder.

### 2.3. Extraction Method

To prepare a hot water extract, 100 mL of water was boiled and poured on 10 g of plant powder. The preparation was closed and set aside to cool. After cooling it was centrifuged and upper layer collected and stored in cold condition for further use. The water extract was subjected to differential solvent extraction with petroleum ether, chloroform, and dichloromethane. The alpha glucosidase inhibitory activity was detected in the chloroform fraction. This fraction was then subjected to silica gel column chromatography with toluene : ethyl acetate gradient. The fractions were collected and evaluated for the inhibitor activity. The 30 : 70 toluene : ethyl acetate fraction showed optimum activity. 10 g of plant powder yielded 70 mg of the column fraction. This CM fraction was used for further studies.

### 2.4. Animals

Adult male A lbino rats weighing between 200 ± 20 g were used. The rats were fed with standard diet purchased from Amruth, India Ltd. Animals were provided with food and water *ad libitum *and maintained at 25–28°C. Diabetes was induced by a single intraperitoneal injection of 70 mg/kg of streptozotocin to overnight fasted rats. The diabetic status of animals was verified after the 14th day of injection. Animals showing blood glucose more than 150 mg/dL were considered as diabetic and used in the experiments. All experiments were carried out on group of six animals. In vitroalpha glucosidase activity was evaluated in quadruplet. 

### 2.5. Effect of CM Fraction on Diabetic Animals in Presence of Disaccharides

The diabetic and normal animals (fasted overnight for 12 h.) were given an oral maltose and sucrose load using gavage tube, and blood glucose was monitored. 2 mg/g body weight of maltose and 4 mg/g body weight of sucrose were orally administered, blood was drawn from tail tip at defined time intervals, and blood glucose was measured using glucometer (Accu check purchased from Roche, India). To study the effect of plant extract, 5.0 mg/kg body weight of the final column fraction was administered 5 minutes before administration of respective sugars and blood glucose monitored. The quantity of the dose was standardized to demonstrate optimum effect.

### 2.6. Isolation of Alpha Glucosidase

Alpha glucosidase was isolated as reported earlier [6]. Briefly normal healthy rats fasting for 20 h were sacrificed by cervical dislocation. The small intestine obtained was flushed several times with ice-cold NaCl and 50 mM (pH 7.0) sodium phosphate buffer. The mucosa was scraped with glass slide on ice-cold glass surface. The obtained material was centrifuged and pellet homogenized in phosphate buffer containing 1% Triton X 100; further cold butanol was added to remove Triton and sample subjected to overnight dialysis. The enzyme thus obtained was used after proper dilution. One unit of enzyme activity (U) is defined as amount of enzyme forming *μ*mole of glucose per min per mL under standard conditions. Assays were performed in quadruplicate.

### 2.7. In Vitro Assay for Alpha Glucosidase and Kinetic Studies

The activity was assayed with 1% starch, 25 mM maltose, 10 mM PNPG as substrate for 30 min and 50 mM sucrose for an hour in presence of phosphate buffer pH 7.2 (100 mM) at 37°C. Reaction was stopped by heat inactivation. Released glucose was quantified using commercial glucose oxidase assay (GOD-PAP Biolab Diagnostics (I) PVT. LTD). The release of PNP from PNPG was measured at 420 nm. Total inhibition for each substrate concentration was determined and IC_50_ value determined at 50% inhibition. Assays were performed in quadruplet. 

For kinetic studies varying concentrations of maltose (10, 15, 20, 25, and 30 mM) were incubated with alpha glucosidase in the absence of inhibitor and with 30 and 50 *μ*g/mL for CM in phosphate buffer pH 7.2 (100 mM) at 37°C, and amount of glucose formed was determined. Data was presented as Lineweaver Burk plots to study the nature of inhibition.

### 2.8. Inhibition Studies for Alpha Glucosidase from Yeast and *α*-Amylase from Aspergillus oryzae

Alpha glucosidase (0.075 units per mL of reaction mixture) (EC 3.4.22.2) from yeast was premixed with and without different concentrations of CM. 5 mM PNPG was added to mixture to start the reaction. PNP formed was measured after 30 minutes of incubation at 420 nm. *α*-amylase (5 mg per mL of reaction mixture) was mixed with and without different concentrations of CM, and 1% starch as a substrate was added. Incubation was carried out for 10 min at 37°C and reaction was stopped by heat inactivation. Reducing sugar was measured by DNSA (Dinitrosalicylic Acid) method.

## 3. Intestinal Absorption Studies

Normal adult albino rats weighing about 200 g were used. After 20 h of fasting, rats were sacrificed by cervical dislocation, and the abdomen was opened by midline incision. The whole small intestine was removed except duodenum and lower part of ileum. The small intestine was washed with chilled saline solution (0.9% w/v NaCl) using syringe several times. Approximately 10 cm of intestinal segments was used for the experiment. One end of the intestinal segment was tied and filled with 0.5 mL of Henseleit bicarbonate buffer (25 mM NaHCO_3_, 118 mM NaCl, 4.7 mM KCl, 1.2 mM MgSO_4_, 1.2 mM CaCl_2,_ and 9.7 mg/L of Na_2_EDTA) containing 100 mM and 200 mM of maltose and sucrose, respectively. The other end was tied, and the sac was placed inside the organ bath containing 20 mL of the same KHB buffer without glucose. Temperature was maintained at 37°C, and proper shaking was ensured. 0.1 mL of 1% of phenol red was added to the intestinal sac in all the experiments to ensure that there is no leakage from the sac. The total incubation period was 40 min. The progress of intestinal absorption was monitored by drawing 0.1 mL of the sample for glucose measurement. Similar experiments were repeated by addition of 0.100 mg of CM and 4 mg, of acarbose to the reaction mixture.

### 3.1. Effect of CM and Acarbose on Oral Load of Maltose and Sucrose

Rats were fasted for 12 h. 2 mg/g body weight and 4 mg/g body weight of maltose and sucrose load were given to diabetic control rats and normal rats. The same experiment was repeated with 5 mg/kg of *C*. *martinii *and 60 mg/kg of acarbose given prior to maltose and sucrose. The level of blood glucose was monitored at 16, 60, and 120 min.

### 3.2. In Vivo Evaluation of Noncompetetitive Inhibiting Action of CM

Rats were fasted for 12 h. 2 mg/g body weight and 3 mg/g body weight of maltose load were given to diabetic control rat. The same experiment was repeated with 5 mg/Kg of *C*. *martinii *and 60 mg/Kg of acarbose given prior to maltose load. The level of blood glucose was monitored at 16, 60, and 120 min.

### 3.3. Data Analysis

All data were expressed as mean ± S.D. for experiment. Statistical significance was analyzed by one-way analysis of variance (ANOVA) with Tukey-Kramer's multiple comparisons test. Readings were considered significant at *P* ≤ 0.05.

## 4. Results

### 4.1. Alpha Glucosidase Inhibition and Measurement of IC_50_ Value

It can be seen from Table 1 that CM can inhibit the maltase activity to the tune of 98% while it is less for sucrase activity. This is in contrast to acarbose which has 15,000 times more affinity for inhibiting the sucrase activity and can also inhibit digestion of starch and maltose [7]. Acarbose demonstrates 100% inhibition for all the substrate.

## 5. Kinetic Studies

**Figure 1:**
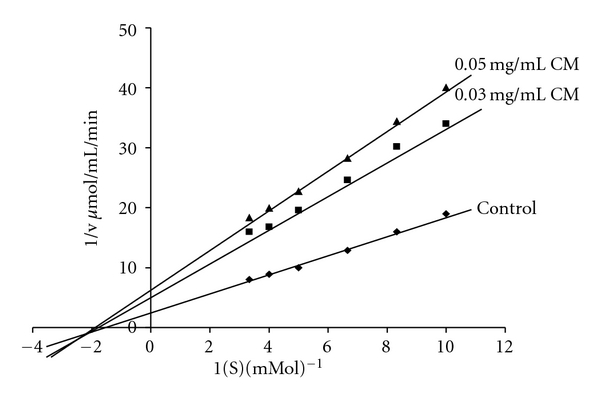
Lineweaver Burk plots for kinetic analysis of alpha glucosidase inhibition by CM at varying concentrations of maltose in presence and absence of different concentrations of plant extract.

The Lineweaver Burk plot (Figure 1) for CM demonstrates a noncompetitive to mixed type of inhibition.

### 5.1. Inhibition of Alpha Glucosidase from Yeast and *α*-Amylase from Aspergillus oryzae

**Figure 2:**
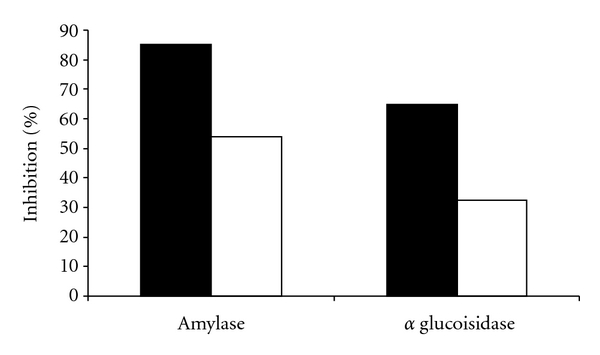
% inhibition by CM (■) and acarbose (□) for alpha Glucosidase from yeast and *α*-amylase from *Aspergillus oryzae *using PNPG and starch as substrates, respectively.

It appears that CM functions as a broad-based inhibitor in that it showed about 73% inhibition for bacterial amylase and almost 85% inhibition of the yeast alpha glucosidase. Acarbose however shows much lower inhibition (Figure 2).

### 5.2. In Vivo Alpha Glucosidase Inhibition Studies

**Figure 3:**
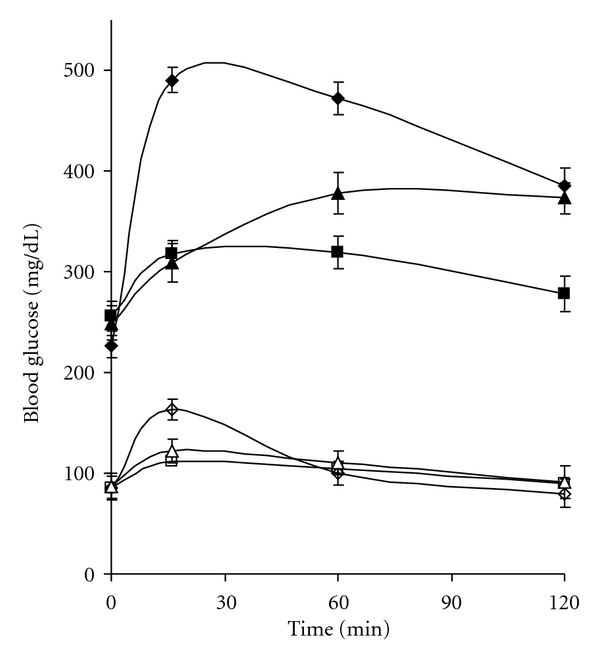
Effect of maltose load (2 mg/g of body weight) on diabetic rats in absence of inhibitor (♦) and in presence of CM (■), acarbose (▲); as well as in normal rats without inhibitor (□) with inhibitor CM (*◊*), acarbose (∆) (*n* = 6, *P < 0.05).

**Figure 4:**
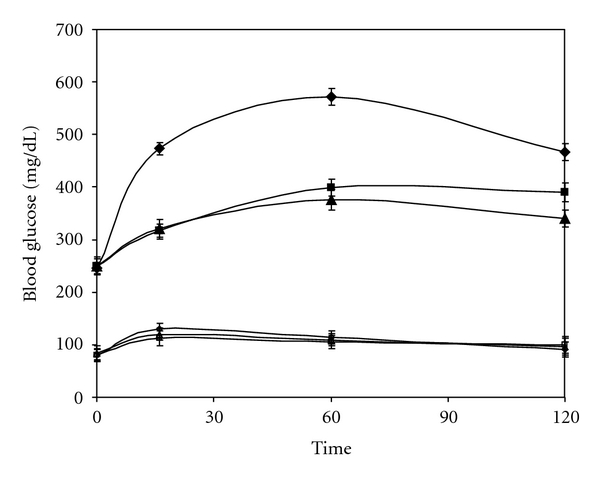
Effect of sucrose load (4 mg/g of body weight) on diabetic rats in absence of inhibitor (♦) and in presence of CM (■), acarbose (▲), as well as in normal rats without inhibitor (□) and with inhibitor CM (*◊*), acarbose (∆) (*n* = 6, *P < 0.05).

The in vivo alpha glucosidase inhibition can be demonstrated by administration of oral maltose and sucrose load. It can be seen from Figure 3 that, as compared to diabetic control, CM shows decrease in the level of blood glucose on maltose load. It should be noted that although CM shows a 98% inhibition in vitro, it demonstrates better lowering of blood glucose, that is, 153 ± 8.6 mg/dL in 2 hours as compared to acarbose. With sucrose as the substrate (Figure 4), acarbose which has very high affinity for sucrose shows lower blood glucose value after two hours. CM which demonstrates about 68% inhibition in vitro shows an increase by 50 ± 7.05 mg/dL as compared to acarbose. In normal animals too, a decrease in the glucose shoot up is seen at 16 min for maltose and sucrose. Administration of CM to normal rats also showed a lowering of postprandial glucose level as compared to the normal rats.

### 5.3. Effect of Plant Extracts on In Vitro Intestinal Digestion of Disaccharides

**Figure 5:**
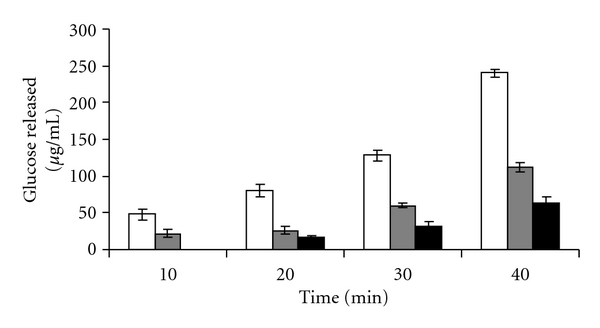
In vitro transport studies using noneverted intestinal sacs with 200 mM sucrose and measurement of glucose released in control (white square), with 100 *μ*gm of CM (grey square), and 4 mg of acarbose (black square) at time intervals of 10, 20, 30, and 40 (*P < 0.05, **P < 0.001).

**Figure 6:**
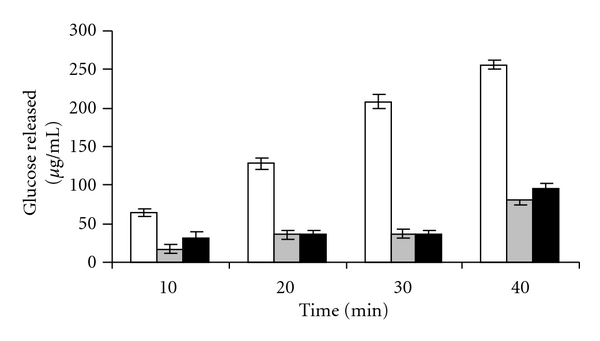
In vitro transport studies using noneverted intestinal sacs with 100 mM maltose and measurement of glucose released in control (white square), with 100 *μ*gm of CM (grey square), and 4 mg of acarbose (black square) at time intervals of 10, 20, 30, and 40 (*P < 0.05, **P < 0.001).

Figure 6 reveals that CM is a good inhibitor of maltose transport showing 69% inhibition as against positive control acarbose which demonstrates a 62% inhibition. Acarbose is a better inhibitor of sucrase activity showing a 73% inhibition (Figure 5). In comparison CM showed 53% inhibition.

### 5.4. In Vivo Noncompetitive Inhibition Study

**Figure 7:**
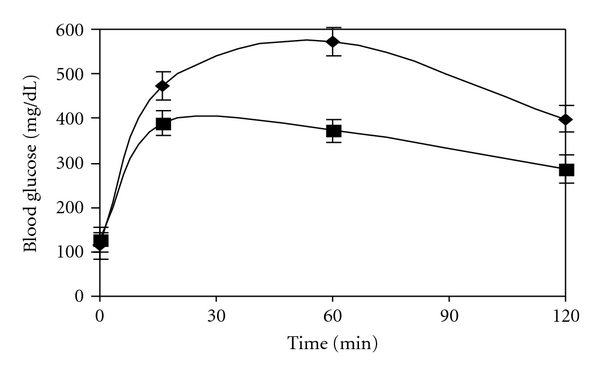
Effect of maltose load of 2 mg/g body weight (■) and 3 mg/g body weight (♦) in diabetic control rats.

**Figure 8:**
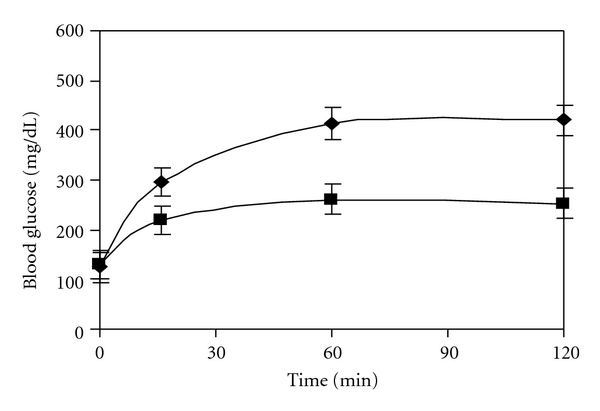
Effect of maltose load 2 mg/g body weight (■) and 3 mg/g body weight (♦) in diabetic control rats in presence of 60 mg/kg acarbose.

**Figure 9:**
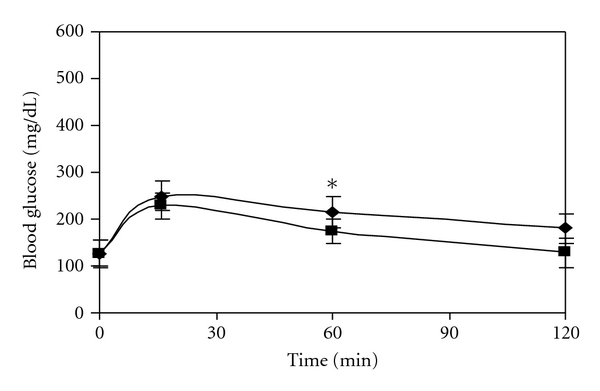
Effect of maltose load 2 mg/g body weight (■) and 3 mg/g body weight (♦) in diabetic control rats in presence of 5 mg/kg of *C*. *martinii*. (*P* < 0.005).

On increasing the maltose load from 2 mg/g to 3 mg/g body weight and measuring the level of blood glucose an increase of 286 ± 12.3 mg/dL and 410 ± 10.3 mg/dL, respectively, was observed after 2 h in diabetic control rats (Figure 7). In case of acarbose though it can be observed from Figure 8 that the 2 h blood glucose was reduced to 250.7 ± 11.3 mg/dL with 2 mg/g body weight load of maltose as compared to diabetic control, the level was found to increase to 375 ± 10.3 mg/dL with 3 mg/g body weight of maltose load. However it is important to note that with CM the value is 128.2 ± 9.12 mg/dL at 2 mg of maltose load/g body weight which marginally increases to 181 ± 11.4 mg/dL at 3 mg of maltose load/g body weight (Figure 9) showing the noncompetitive mode of inhibition by CM even under in vivo conditions.

## 6. Discussion

The use of alpha glucosidase inhibitor treatment in control of rise in the postprandial glucose level is desirable as it constitutes a noninvasive mechanism for controlling hyperglycemia. The STOP NIDDM study published in 2002 has revealed the advantage of alpha glucosidase inhibitors in prevention of progress of potential diabetic patients into Type II diabetic patients in addition to controlling the risk of cardiovascular damage [8]. Diabetic patients generally suffer from hyperglycemic shoot up after meals which take almost 4-5 hours to reduce back to the original glucose level. The shoot up is attributed to increased disaccharidases activity by 1.5-fold [9] and glucose transporters GLUT2 and SGLT1 activity by almost 3-4-fold in diabetic animals as compared to normal [10]. The increased glucose level for prolonged time leads to nonspecific glycation of proteins initiating a cascade of secondary complications [11]. Hence, control of postprandial glucose levels would be valuable in prevention of secondary complications of diabetes. 

*Cymbopogon martinii *is routinely used in the Indian Ayurvedic Medicine System for a long period, and no known toxicity reports are available neither have we observed any toxic effects in the experimental animals, even at a dose 10 times higher than reported in this paper. The plant appears to be very good inhibitor of maltase activity while it exhibits 68% inhibition of sucrase activity. The mechanism is distinct from acarbose which is a strong inhibitor of sucrose [12] (Enc et al. 2001). CM being more of a noncompetitive type inhibitor of alpha glucosidase would bind to the enzyme at a region other than the active site and may not be affected by higher concentration of the substrate as against acarbose which is a competitive inhibitor [7]. We have also observed inhibition of alpha glucosidase by *Tinospora cordifolia *stem extract to be of noncompetitive type [6]. A case study on Sri Lankan plant *Cassia auriculata *which has similar glucosidase inhibitory activity shows reduction in blood glucose on maltose load but no such action with sucrose load [8]. In case of *Punica granatum *the flower extract is reported to have sucrase inhibiting activity even more potent than *Salacia oblonga *which is known to possess potent sucrase inhibition action, however no action of inhibition on maltose is mentioned [13]. Acarbose shows milder inhibition of both the alpha glucosidase from yeast and bacteria [14]. On the other hand several plant glucosidases reported to have noncompetitive inhibition show substantial inhibition of both these enzymes [8, 14]. The ability to bind to sites other than the active site probably gives them a broader specificity of inhibition as is also observed in CM. 

A reduction in intestinal glucose absorption by perfusion of glucose in rat intestine with plant extract of *Plantago ovata *is suggested to be due to increased gastric motility by the high fiber content and not due to inhibition of the transporter [15]. To ensure that reduction in blood glucose was due to inhibition of alpha glucosidase activity, in vitro intestinal absorption was studied. CM appears to be a better inhibitor of maltase activity than acarbose although the latter has higher sucrase inhibiting action. However, as the regular diet is rich in starch than sucrose, CM apparently has better advantage in treatment of diabetes. Unlike acarbose which is reported to stimulate GLP-1 secretion leading to insulin release and helping in reduction in postprandial glucose level [12] no increase in insulin level was evident with CM revealing that their antihyperglycemic action is predominantly due to inhibition of alpha glucosidase (results not shown). It is interesting to note that although acarbose demonstrates better IC_50_ value and 1000% inhibition of maltose activity in vitro CM shows better inhibition in vivo and presents shoot up in blood glucose value for prolonged period which is advantages in treatment of diabetes. It should also be noted from Figures 3 and 4 that inhibitory action of CM is at a concentration of 5.0 mg/kg body weight as against 60 mg/kg body weight for acarbose. On further purification of the active principle the required inhibitory concentration of the plant components will be even lower, demonstrating their potent alpha glucosidase inhibitory action. 

The major advantage of CM over acarbose, apart from its higher maltase inhibitory action, is that it demonstrates noncompetitive mode of inhibition. It is evident that, with higher feed, higher concentration of acarbose would be needed to show the same effect; the same is not the case with CM which is still effective at lower concentration. The major side effects of acarbose such as flatulence, intestinal disturbances would be enhanced with higher concentration. 

In conclusion we report for the first time, *Cymbopogon martinii *commonly found grass everywhere with no reported toxicity to possess excellent alpha glucosidase inhibition activity. The effect is more pronounced for CM as compared to acarbose which is currently the most widely used alpha glucosidase inhibitor for treatment of diabetes. Thus CM can generate better lead molecule as alpha glucosidase inhibitor for treatment of diabetes mellitus particularly in control of postprandial glucose level.

## Figures and Tables

**Table 1:** Inhibition of intestinal *α*-glucosidase by CM with different substrates. IC_50_ value for CM is 40 *μ*gm/mL for starch, maltose, and PNPG, while it is 60 *μ*gm/mL for sucrose. (Acarbose shows 100% inhibition for all substrates. IC_50_ 60 *μ*gm/mL for starch, maltose and PNPG and 30 *μ*gm/mL for sucrose).

	*Cymbopogon martini*		Acarbose	
Substrate	IC_50 _ (*μ*g/mL)	% inhibition	IC_50 _ (*μ*g/mL)	% inhibition
1% starch	40	98	60	100
Maltose	40	98	60	100
PNPG	40	98	60	100
Sucrose	60	68	30	100
